# Case Report: Mutant *SCN9A* Susceptible to Charcot Neuroarthropathy in a Patient With Congenital Insensitivity to Pain

**DOI:** 10.3389/fnins.2021.697167

**Published:** 2021-07-14

**Authors:** Xiao-hui Xie, Jian-guang Tang, Zhong-hua Liu, Shui-jiao Peng, Zhuang-zhuang Yuan, Heng Gu, Yi-qiao Hu, Zhi-ping Tan

**Affiliations:** ^1^Clinical Center for Gene Diagnosis and Therapy, Department of Cardiovascular Surgery, The Second Xiangya Hospital of Central South University, Changsha, China; ^2^Department of Neurology, The Second Xiangya Hospital of Central South University, Changsha, China; ^3^The National and Local Joint Engineering Laboratory of Animal Peptide Drug Development, College of Life Sciences, Hunan Normal University, Changsha, China; ^4^Department of Cell Biology, School of Life Sciences, Central South University, Changsha, China

**Keywords:** Charcot neuroarthropathy, bone destruction, joint destruction, congenital insensitivity to pain, *SCN9A*

## Abstract

Charcot neuroarthropathy is a systemic disease with pathological changes in the musculoskeletal system, which leads to fractures, dislocations, and deformities involving multiple bones and joints, particularly those of the feet. While the common underlying cause of Charcot neuroarthropathy is diabetes mellitus, it is also associated with congenital insensitivity to pain (CIP). CIP is a rare disorder caused by loss-of-function mutations in *SCN9A* encoding Nav1.7. In this study, we report a patient with CIP from a consanguineous family susceptible to Charcot neuroarthropathy with a novel *SCN9A* mutation. This report involves the case of a middle-aged man who suffered from CIP, had repeated painless fractures, and developed bone and joint destruction. The physical and radiological examinations revealed that multiple joints were swollen and deformed, and soft-tissue trauma was evident. We identified a novel homozygous *SCN9A* mutation (p.Cys1339Arg) by whole-exome sequencing (WES), which was verified using Sanger sequencing. In addition, the wild-type (WT) and mutated p. Cys1339Arg were assessed in HEK293 cells expressing Nav1.7, and the results showed that p. Cys1339Arg almost abolished the Nav1.7 sodium current. In conclusion, Charcot neuroarthropathy associated with CIP demonstrated a wider spectrum of Charcot neuroarthropathy than was previously recognized or documented. In addition, this finding is conducive to understanding the critical amino acids for maintaining the function of Nav1.7, thus contributing to the development of Nav1.7-targeted analgesics.

## Introduction

Charcot neuroarthropathy is a systemic disease with pathological changes in the musculoskeletal system, which lead to fractures, dislocations, and deformities involving multiple bones and joints, particularly those of the foot (Dodd and Daniels, 2018). In 1868, the famous neuropathologist Jean-Martin Charcot first described the condition of “Charcot neuroarthropathy,” based on his clinical observation of a patient with neurosyphilis and peripheral joint destruction (Hubault, 1982). Nowadays, with the increase in the incidence of diabetes, the common underlying cause of Charcot neuroarthropathy is diabetes mellitus (Watts et al., 2016). However, Charcot neuroarthropathy is also associated with congenital insensitivity to pain (CIP), which has rarely been reported (Cassidy and Shaffer, 2008).

Pain sensation is an essential protective mechanism to minimize tissue and cellular damage. CIP, a rare autosomal-recessive disorder, is characterized by the lack of protective mechanisms of pain against noxious stimuli, predisposing patients to self-mutilations, burns, and painless fractures (Waxman et al., 2014). Anosmia is also a common clinical feature of patients with CIP. Cox et al. (2006) reported that loss-of-function mutations in *SCN9A*, which encodes the voltage-gated sodium channel (VGSC) Nav1.7, caused congenital inability to experience pain and anosmia. While self-mutilation and anosmia are common, Charcot joints are not common features of Nav1.7-related CIP. The progression of Charcot neuroarthropathy is generally attributed to repetitive microtrauma in patients with impaired pain sensation and proprioception.

The development of Charcot neuroarthropathy in patients with CIP is rare, with few cases reported in the literature. Both clinical and genetic observations of Charcot neuroarthropathy are significant in improving the understanding of this condition. In this report, we have described a Chinese Han man who developed Charcot neuroarthropathy secondary to CIP. We identified a novel *SCN9A* mutation by whole-exome sequencing (WES), which was verified using Sanger sequencing. In addition, we detected the changes in electrophysiological activity using whole-cell patch-clamp recordings.

## Case Description

**FIGURE 1:**
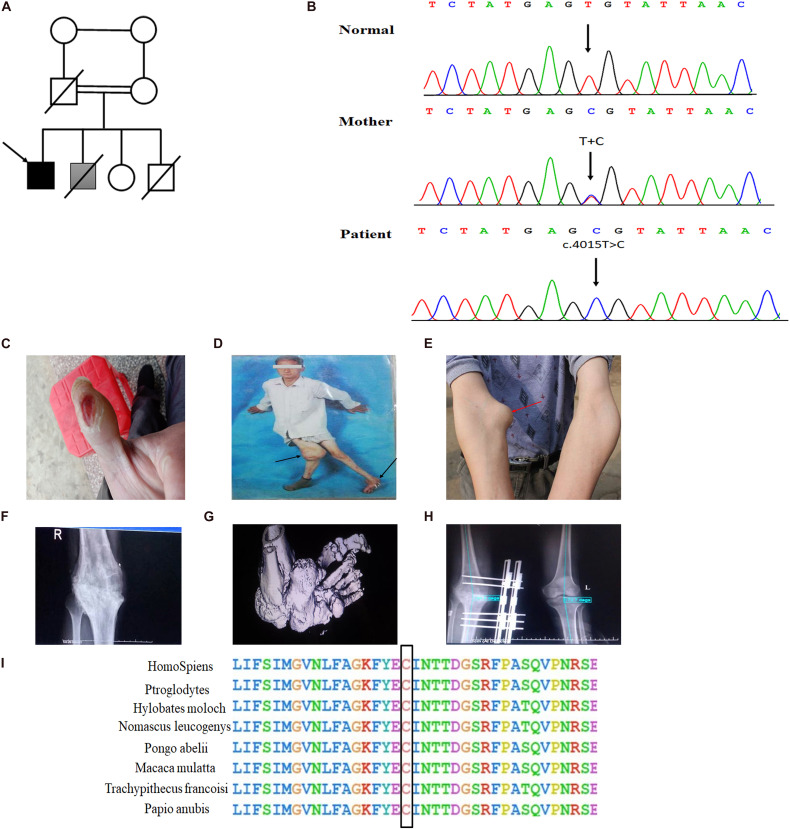
Novel homozygous *SCN9A* mutation and the clinical phenotypes in the proband with Charcot neuroarthropathy. **(A)** Family pedigree. The proband is indicated by a black arrow. The shaded box represents a suspect patient who is presented with Charcot neuroarthropathy. The genetic relationship of parents is them being first cousins. **(B)** DNA sequence electropherograms demonstrating c.4015T > C (p. Cys1339Arg) homozygous mutation in the proband. **(C)** Burns on the finger by a lit cigarette. **(D,E)** Arrows of different colors represent swollen and deformed joints. **(F)** Plain radiographs of the right knee joint showed narrowing of the right knee joint space, blurring of the joint surface, bone destruction, and uneven density. The knee is swollen, distorted, and painless. **(G)** The three-dimensional CT of the left foot showed that the structures of the left ankle joint, the left tarsometatarsal joint, and the intertarsal joint were disordered, and the left ankle showed varus and subluxation. **(H)** The postoperative x-ray examination of bilateral knee joints showed that the internal fixation device was in place after the correction of the valgus of the right knee. **(I)** Conservation of residues Cys1339 among different species.

In the present consanguineous Chinese family, the proband was a 42-year-old man who presented with Charcot neuroarthropathy. He was the fourth child of parents with normal pregnancy and childbirth (Figure 1A). Although his older brother had similar symptoms, we were unable to collect the data because he died from a severe accident in the previous year. The proband had no history of diabetes mellitus, hypertension, and syphilis, which are common risk factors for Charcot neuroarthropathy. However, he reported that he had been unable to respond to pain since birth. When he was 1 year old, he often bit his fingers without any sensation of pain; later, his parents noticed that he was incapable of experiencing pain. When he was 10 years old, he repeatedly suffered painless fractures and got burned unknowingly. When he started smoking, he often burned his fingers with lit cigarettes but did not experience pain (Figure 1C). Furthermore, he did not experience pain due to extreme heat or cold, although he could distinguish between hot and cold sensations. However, the proband had a complete sense of smell as he could distinguish smells of water, alcohol, or white vinegar. In his 30 s, he developed multiple joint swelling, instability, and deformities caused by repetitive microtrauma and painless fractures (Figures 1D,E). Bone and joint destruction induced a severe negative impact on the quality of his life.

Detailed physical examination revealed swelling in the right knee, left ankle, and right elbow joint; reduced right knee flexion; valgus deformity; and left ankle varus. Skin ulcers were noted on the right knee and on the lateral side of the left ankle (Figure 1D). A neurological examination indicated normal cognitive function (Table 1). In addition, radiography of the right knee joint showed narrowing of the right knee joint space, blurring of the joint surface, bone destruction, and uneven density (Figure 1F). Three-dimensional CT scan of the left foot demonstrated that the structures of the left ankle joint, left tarsometatarsal joint, and the intertarsal joint were disordered, and the left ankle exhibited varus and subluxation (Figure 1G). After various evaluations, the proband underwent focus debridement and fusion external fixation of the left knee. Postoperative radiographic examination of the bilateral knee joints revealed that the internal fixation device was in place after correction of the valgus of the right knee, and there were no apparent signs of loosening or fracture (Figure 1H). During postoperative, the proband recovered well and could walk with crutches.

**TABLE 1:** Summary of clinical findings in the proband.

Clinical features	

Age	Symptoms
1	Self-mutilation (bit fingers)
10	Painless injuries (fractures, burns)
30	Charcot joints
42	Bone and joint destruction

**Examinations**	

**Examination**	**Y or N**

Anosmia	N
Normal cognitive development	Y
Joint deformity	Y
Soft-tissue ulcer	Y

*N, no; Y, yes.*

### Mutation Analysis

Whole peripheral blood samples were collected and stored in ethylenediaminetetraacetic acid (EDTA) tubes. Genomic DNA was extracted using a QIAamp DNA Blood Mini Kit (250) (Qiagen, Valencia, CA, United States). WES was performed at the Berry Genomics bioinformatics institute (Beijing, China). The exomes were captured using Agilent SureSelect Human All Exon V6 kits and sequenced on a NovaSeq 6000 system (Illumina, Inc., San Diego, CA, United States). The WES data were filtered by three criteria: (1) variations outside the coding regions (e.g., intergenic, intronic, and untranslated regions) and synonymous mutations were excluded; (2) high allele frequencies relative to population databases (>0.01%) (e.g., 1,000 Genomes Project, ESP, and gnomAD) were excluded; and (3) prediction of a deleterious functional effect by bioinformatics programs (e.g., MutationTaster; Polyphen2; Combined Annotation Dependent Depletion, CADD; and SIFT), loss of function, and deleterious mutations were considered.

Sanger sequencing was performed to verify the identified variant. The primers were designed by Primer Quest Tool (F:5′-GCTGGTTGGTTTGATGTCTTAG, R:5′-GGAACTTGA CTTGCAGGAAAC). The PCR conditions were set as follows: 95°C for 30 s, 56°C for 30 s, and 72°C for 1 min, for a total of 35 cycles using T3 Super PCR Mix (TSINGKE, Beijing, China). The PCR products were electrophoresed on a 2% agarose gel. The PCR fragments were subsequently cut, and the purified fragments were sequenced on a 3730 XL sequencer (Applied Biosystems).

### Identification of a Novel *SCN9A* Mutation

Whole exome sequencing, a high-throughput sequencing technique, was performed to generate 12 Gb of data with 99% coverage and a depth of >100X. After filtration of the WES data, we finally identified a missense homozygous mutation (c.T4015C; Cys1339Arg) in exon 21 of *SCN9A*, which was further confirmed by Sanger sequencing (Figure 1B). The variant was predicted to be “disease-causing” by MutationTaster and was not found in the 1,000 Genome Browser, ExAC Browser, Exome Variant Server, or in 500 unrelated ethnically matched healthy controls. The controls were individuals presenting for routine health checkups or volunteers without similar symptoms or any positive family history of musculoskeletal system disorders and CIP.

**FIGURE 2:**
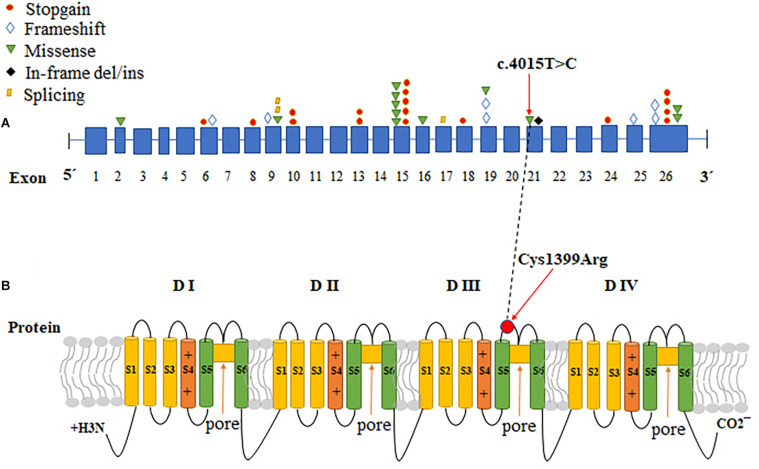
The CDS sequence and protein structure pattern of *SCN9A*. **(A)** A variety of mutations in different exons in *SCN9A* cause CIP. The red circles: stopgain; blue diamonds: frameshift; green inverted triangles: missense; black diamonds: in-frame del/ins; yellow parallelograms: splicing. **(B)** Schematic representation of Nav1.7, the voltage-gated sodium channel (VGSC) a-subunit encoded by *SCN9A*, and the locations of the identified mutation. *SCN9A* encodes a plasma membrane protein, Nav1.7, which consisted of four similar domains with each domain comprising six a-helical transmembrane segments (labeled 1–6). Transmembrane segments 5 and 6 are the pore-lining segments and the voltage sensor is located in transmembrane segment 4 of each domain (depicted by a plus symbol). The red arrows indicate the location of the missense mutation identified in this study. The black dotted line correlates the change of CDS with protein change in SCN9A.

The Nav1.7 mutation was predicted to result in amino acid substitutions p. Cys1339Arg in the extracellular linker joining S5 and S6 of domain III, outside the end of S5 (Figure 2B). Multiple alignments of *SCN9A* orthologs in other animal species indicated that the amino acid at position 1339 was highly conserved (Figure 1I).

### Electrophysiological Properties of Mutant Nav1.7 Channels

To determine the genotype and clinical phenotype correlation in the patient, we investigated the effect of the variation on the electrophysiological activity of Nav1.7 by performing whole-cell patch-clamp recordings in HEK cells inward sodium channel traces of wild-type (WT) and p.C1339R mutant Nav1.7 channels. First, to construct the Nav1.7 expression plasmid, the cDNA of human Nav1.7 (NM_002977.3) was subcloned into the pcDNA3.1mod vector, and mutations (c.4015T > C, p. Cys1339Arg) (referred to as C1339R) were introduced using overlap extension PCR with primers and verified by Sanger sequencing. There was no other modification of the pcDNA3.1mod vector. HEK293 cells were cultured in 3.5 cm plastic dishes (NEST) in Dulbecco’s Modified Eagle Medium (Invitrogen, Beijing, China) supplemented with 10% fetal bovine serum, glutamine, and Penicillin-Streptomycin solution (Invitrogen). In addition, cells were grown under standard conditions (37°C, 5% CO_2_) for approximately 12 h. Subsequently, cells were co-transfected with WT or mutant *SCN9A* plus *SCN1B* plus *SCN2B* plus *EGFP* using the Lipofectamine 2000 transfection reagent (Invitrogen, Carlsbad, CA, United States) according to the instructions of the manufacturer and incubated for 24 h.

**FIGURE 3:**
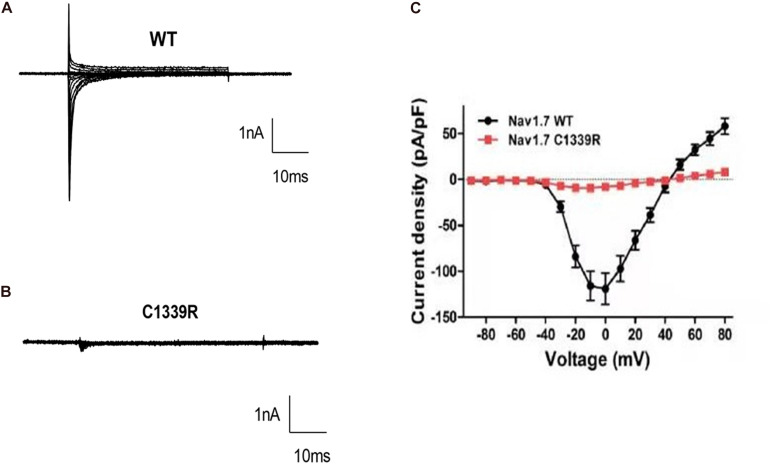
Biophysical characterization of WT and mutant Nav1.7 channels in HEK293 cells. **(A,B)** Representative inward current traces were recorded from HEK293 cells expressing WT, and mutant p. Cys1339Arg Nav1.7 channels, respectively. **(C)** Current-voltage relationship of WT and mutant p. Cys1339Arg channels. Peak current density normalized to membrane capacitance is presented as the mean ± SEM.

Finally, custom-designed Exclamp software was used at 22 ± 2°C. The recording platform was EPC10 USB Amplifier (HEKA), and the control software was PatchMaster (HEKA). The extracellular bath solution included 140 mM NaCl, 5 mM KCl, 2 mM CaCl_2_, 10 mM HEPES, 1 mM MgCl_2_, and 10 mM glucose (pH 7.4, adjusted with NaOH). The pipette solution contained the following in mM concentrations: 105 CsF, 35 NaCl, 10 HEPES, and 10 EGTA (pH 7.4, adjusted with CsOH). The glass electrode was created using a microelectrode glass capillary (Wuhan Microprobe Scientific Instrument Co., Ltd., China, PC-10 electrode drawing instrument) using a two-step method, and the resistance of the electrode to water was controlled at 2–3 mΩ. An activation protocol was used whereby cells were voltage-clamped at a holding potential of −90 mV, followed by a 50 ms step to the activating step (−90 mV to +80 mV, 10 mV steps). The peak inward current at each activating step was quantified and presented as current-voltage curves. Finally, the data were analyzed using Igor pro6.10A and GraphPad Prism 8. Representative inward sodium channel traces of WT and p.C1339R are shown in Figures 3A,B. The results showed that the average peak current density of the p.C1339R channels was almost abolished compared with that of the WT channels (−9.3 ± 1.5 pA/pF, *N* = 10. vs. −119.1 ± 16.0 pA/pF, *N* = 22, *P* < 0.05) (Figure 3C).

## Discussion

In this study, we identified a novel homozygous missense mutation, Cys1339Arg, in *SCN9A* in the proband susceptible to Charcot neuroarthropathy by WES, which was verified by Sanger sequencing. The mutation was located in the extracellular linker joining S5 and S6 of domain III, outside the mouth of the pore. In addition, the patch-clamp recordings revealed that p.Cys1339Arg of Nav1.7 is a non-functional channel because of the almost non-existent inward charge density.

The proband from a consanguineous family exhibited CIP, but not anosmia, and presented with bone and joint destruction. Although CIP is most common in childhood (Shorer et al., 2014), this is a rare case of Charcot neuroarthropathy in a middle-aged patient with congenital inability to experience pain. The proband was diagnosed with Charcot neuroarthropathy based on thorough medical history and examinations, including clinical features, such as the loss of pain sensation; the presence of ulcers; and a warm and swollen foot, ankle, and knee (Figures 1C–E). The physical and radiological examinations showed that multiple joints were swollen and deformed, and soft-tissue trauma was evident (Figures 1D–H). While other causes of Charcot neuroarthropathy cannot be ruled out, CIP is likely to be a contributing factor in this case.

Previous studies emphasized that diabetes mellitus is the common underlying cause of Charcot neuroarthropathy, and nearly 1% of patients with diabetes presented neuropathic changes in the foot (McInnes, 2012); however, CIP is a rare risk factor of neuropathic arthropathy. The proband did not receive careful supervision from his parents when he was diagnosed with CIP in his childhood, and repetitive painless fractures are one of the factors that induce Charcot neuroarthropathy. To further explore the relationship between CIP and Charcot neuroarthropathy, we collected clinical data and carried out WES and patch-clamp recordings. As a result, a novel missense homozygous *SCN9A* variant (c.4015T > C; p.Cys1339Arg) was identified and predicted to be a disease-causing variant by MutationTaster and CADD. In addition, the electrophysiological tests showed that p.Cys1339Arg of Nav1.7 almost abolished current density. Considering that the Nav1.7 plays a vital role in signal pathways in nociceptive neurons (Haberberger et al., 2019), the mutant Nav1.7 completely abolishes its function as a VGSC in nociceptors. Therefore, the clinical feature of insensitivity to pain in the proband seemed well explained. Charcot neuroarthropathy may attribute to long-term impaired pain sensation, unnoticed damage, and repetitive microtrauma to the weight-bearing joints (Callaghan et al., 2015).

*SCN9A*, found at 2q24.3, is a 26-exon gene that encodes the VGSC Nav1.7, which is highly expressed in peripheral sensory neurons, most notably in nociceptive small-diameter DRG neurons and sympathetic ganglion neurons (Klugbauer et al., 1995; Toledo-Aral et al., 1997; Rush et al., 2006). VGSCs comprises pore-forming α-subunits and auxiliary β-subunits. Each α-subunit consists of four homologous domains (DI-DIV), with each containing six transmembrane segments (S1–S6), connected by intracellular loops (L1, L2, and L3) and cytoplasmic N- and C-terminal regions (Catterall, 2000). Mutations that cause a gain of function in NaV1.7 have been shown to cause autosomal-dominant pain disorders, such as primary erythermalgia. Conversely, loss-of-function mutations in *SCN9A* contribute to CIP (Klein et al., 2013). To date, more than 100 mutations in *SCN9A* have been reported, but only 42 have been associated with CIP (Baker and Nassar, 2020; Supplementary Table 1 and Figure 2A). Among the *SCN9A* mutations identified in CIP patients, most were non-sense, frameshift, or splicing mutations, all of which produced non-functional and truncated Nav1.7 proteins; however, we identified a novel homozygous missense mutation (c.4015T > C, p. Cys1339Arg) in *SCN9A* in a patient with CIP, which abolished the current density and the Nav1.7 function. The variant site was localized in exon 21 of *SCN9A*, and the protein change was located in the extracellular linker joining S5 and S6 of domain III, which has not been reported previously (Figure 2). S5-P, P loop, and P-S6 are vital for the selective permeation of sodium ions (Catterall, 2017). Residue Cys1339 mapped to the S5-P, domain III of Nav1.7, is highly conserved among different species (Figure 1I) and plays a role in filtering sodium ions.

Loss-of-function mutations in the VGSC Nav1.7 cause CIP in human, making Nav1.7 an important target for novel analgesics. For humans, in most patients with CIP, the perception of non-noxious touch and warmth is not affected, whereas the perception of noxious heat, pressure, and injury pain is completely lost. Therefore, loss of Nav1.7 did not lead to lethality or any disability (Zufall et al., 2012). Selective Nav1.7 channel blockers, as potential painkillers with improved safety and reduced unwanted side effects, will be increasingly favored by doctors and patients. For mice, a study indicated that mice with selective knockout of Nav1.7 in nociceptive neurons showed deficits in acute and inflammatory pain sensation, whereas a global knockout (KO) of Nav1.7 was neonatal lethal because the animals are anosmic and cannot feed (Nassar et al., 2004). Indeed, a global KO mouse was generated on a different genetic background that partially alleviated anosmia and allowed the mice to survive, which provides a powerful tool for pre-clinical experiments in the analgesic field (Grubinska et al., 2019). To determine the maximum effect of a theoretically perfect selective Nav1.7 inhibitor, a research team generated a tamoxifen-inducible KO mouse model that enabled the genetic deletion of Nav1.7 in adult mice (Shields et al., 2018). The selective blocker site of the NaV1.7 channel, with minimal side effects, is a significant target for analgesic drugs. According to the structure of Nav1.7, S4 carries gating charges in the sliding helix or helical screw model of voltage sensing. Amino acid residues in the short segments between S5 and S6 form the receptor site for the pore blocker tetrodotoxin (Catterall, 2017). The novel mutation p.Cys1339Arg is located in the extracellular linker joining S5 and S6 of domain III, outside the mouth of the pore-forming region, which is a significant part of Nav1.7. In addition, the patient in this study with the p.Cys1339Arg variant in Nav1.7 developed Charcot neuroarthropathy in conjunction with CIP, which may arise attention in pain drugs development programs.

In summary, the novel missense homozygous variant p.Cys1339Arg is a loss-of-function mutation in Nav1.7, which constitutes a novel mutation susceptible to neuroarthropathy. Charcot neuroarthropathy associated with CIP demonstrated a wider spectrum of Charcot arthropathy of the foot and ankle than that which has previously been recognized or documented. In addition, these findings help in understanding the critical amino acid for maintaining the function of Nav1.7, thus contributing to the development of Nav1.7-targeted analgesics.

## Data Availability Statement

The datasets presented in this study can be found in online repositories. The names of the repository/repositories and accession number(s) can be found below: ClinVar, SCV001572593.

## Ethics Statement

The studies involving human participants were reviewed and approved by the Ethics Committee of The Second Xiangya Hospital of Central South University. The patients/participants provided their written informed consent to participate in this study. Written informed consent was obtained from the individual(s) for the publication of any potentially identifiable images or data included in this article.

## Author Contributions

Z-PT designed the overall study and performed the data analysis. X-HX processed the WES data, validated the mutation, and drafted the manuscript. J-GT enrolled the subjects. Z-HL and S-JP carried out the electrophysiological analysis. Z-ZY analyzed the data and edited the manuscript. Y-QH and HG conducted the cell culture and transfection. All authors read and approved the final version of the manuscript, contributed to the article, and approved the submitted version.

## Conflict of Interest

The authors declare that the research was conducted in the absence of any commercial or financial relationships that could be construed as a potential conflict of interest.

## Supplementary Material

Supplementary Table 1 *SCN9A* mutations that cause congenital insensitivity to pain (CIP): in order of their publications.

## Abbreviations

CIP: congenital insensitivity to pain
VGSC: voltage-gated sodium channel
WES: whole-exome sequencing
PCR: polymerase chain reaction
WT: wild-type
DRG: dorsal root ganglion.
