# Supplementary material for: Case Report: Mutant SCN9A Susceptible to Charcot Neuroarthropathy in a Patient With Congenital Insensitivity to Pain
Source: Front Neurosci. 2021 Jul 14;15:697167. doi: 10.3389/fnins.2021.697167 (PMC8317969; doi:10.3389/fnins.2021.697167)
Supplement: Supplementary Table 1 — SCN9A mutations that cause congenital insensitivity to pain (CIP): in order of their publications. [file Table_1.docx]

**
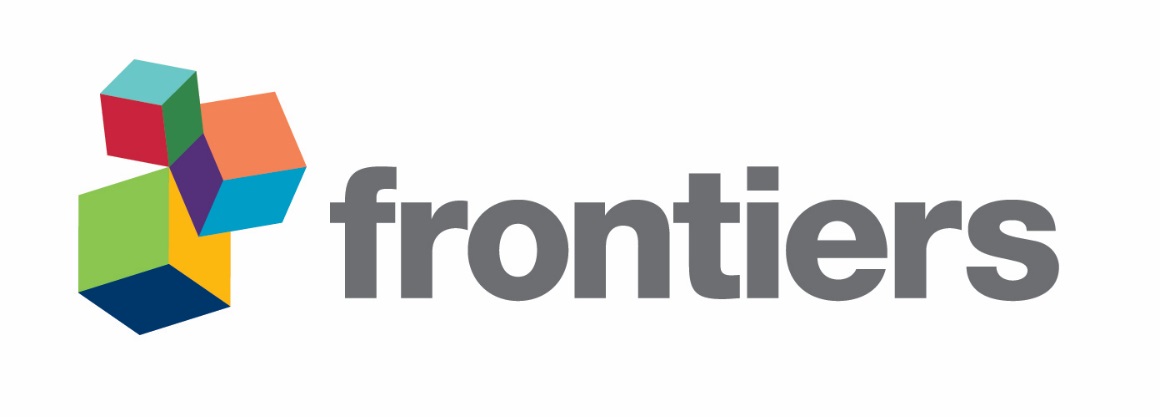
**

**Supplementary Table 1** *SCN9A* mutations that cause cogenital insensitivity to pain (CIP) in order of their publications

| Mutation | Protein change | Exon | Mutation type | Reference |
| --- | --- | --- | --- | --- |
| c.296G>A | p. R99H | E2 | Missense | (Sun et al., 2020) |
| c.829C>T | p. R277X | E6 | Stopgain | (Goldberg et al., 2007) |
| c.828delGT | p. F276Lfs*6 | E6 | Frameshift | (Nilsen et al., 2009) |
| c.984C>A | p. Y328X | E8 | Stopgain | (Goldberg et al., 2007) |
| c.IVS8-2A>G |  | E9 | Splicing | (Klein et al., 2013) |
| c.1126A>C | p. K376Q | E9 | Missense | (Shorer et al., 2014) |
| c.1124delG | p. G375Afs | E9 | Frameshift | (Shorer et al., 2014) |
| c.1108-2A>G |  | E9 | Splicing | (Marchi et al., 2018) |
| c.1567C>T | p. R523X | E10 | Stopgain | (Kurban et al., 2010) |
| c.1376C>G | p. S459X | E10 | Stopgain | (Cox et al., 2006) |
| c.2298delT | p. I767X | E13 | Stopgain | (Cox et al., 2006) |
| c.2076-2077insT | p. E693X | E13 | Stopgain | (Goldberg et al., 2007) |
| c.2691G>A | p. W897X | E15 | Stopgain | (Cox et al., 2006) |
| c.2687G>A | p. R896Q | E15 | Missense | (Marchi et al., 2018) |
| c.2488C>T | p. R830X | E15 | Stopgain | (Goldberg et al., 2007) |
| c.2796A>C | p.M932L | E15 | Missense | (Yuan et al., 2011) |
| c. 2697G>A | p.M899I | E15 | Missense | (Yuan et al., 2011) |
| c.2575C>T |  | E15 | Stopgain | (Nilsen et al., 2009) |
| c.2755G>T | p. E919X | E15 | Stopgain | (Peddareddygari et al., 2014) |
| c.2720G>A | p. R907Q | E15 | Missense | (Remacle et al., 2015) |
| c.2749T>G | p. W917G | E15 | Missense | (Sun et al., 2020) |
| c.2488C>T | p.830X | E15 | Stopgain | (Goldberg et al., 2007) |
| c.3312G>T | p. V1104L | E16 | Missense | (Yuan et al., 2011) |
| c.3319-2A>G |  | E17 | Splicing | (Rajasekharan et al., 2017) |
| c.3567_3567delC | p. M1190X | E18 | Stopgain | (Sawal et al., 2016) |
| c.3703-3713del | p. I1235LfsX2 | E19 | Frameshift | (Goldberg et al., 2007) |
| c.3600delT | p. F1200Lfs | E19 | Frameshift | (Goldberg et al., 2007) |
| c.3707C>A | p.A1236E | E19 | Missense | (Emery et al., 2015) |
| c.4015T>C | p.C1339R | E21 | Missense | This study |
| c.4108_4122delCGATGGAAAAACCTG | DeltaR1370-L1734 | E21 | In-frame del | (Cox et al., 2010) |
| c.4462C>T | p. R1488X | E24 | Stopgain | (Goldberg et al., 2007) |
| c.4474_delA | p. I1493SfsX8 | E25 | Frameshift | (Cox et al., 2010) |
| c.5067G>A | p. W1689X | E26 | Stopgain | (Goldberg et al., 2007) |
| c.4975A>T | p. K1659X | E26 | Stopgain | (Goldberg et al., 2007) |
| c.5155T>C | p.C1719R | E26 | Missense | (Staud et al., 2011) |
| c.5323T>A | p. W1775R | E26 | Missense | (Emery et al., 2015) |
| c.5492T>G | p. L1831X | E26 | Stopgain | (Emery et al., 2015) |
| c.4795C>T | p. R1599X | E26 | Stopgain | (Mansouri et al., 2014) |
| c.5318delA | p. FS1773 | E26 | Frameshift | (Ramirez et al., 2014) |
| c.5463dupT | p.G1822 fs | E26 | Frameshift | (Rajasekharan et al., 2017) |
| Splice-junction mutation intron 23-24 |  | Intron23-24 | Splicing | (Goldberg et al., 2007) |

Individual mutations are provided in the table and numbered according to accession number NM_002977 in GenBank

Cox, J.J., Reimann, F., Nicholas, A.K., Thornton, G., Roberts, E., Springell, K., et al. (2006). An SCN9A channelopathy causes congenital inability to experience pain. *Nature* 444(7121)**,** 894-898. doi: 10.1038/nature05413.

Cox, J.J., Sheynin, J., Shorer, Z., Reimann, F., Nicholas, A.K., Zubovic, L., et al. (2010). Congenital Insensitivity to Pain: Novel SCN9A Missense and In-frame Deletion Mutations. *Human Mutation* 31(9)**,** E1670-E1686. doi: 10.1002/humu.21325.

Emery, E., Habib, A., Cox, J., Nicholas, A., Gribble, F., Woods, C., et al. (2015). Novel SCN9A mutations underlying extreme pain phenotypes: unexpected electrophysiological and clinical phenotype correlations. 35(20)**,** 7674-7681. doi: 10.1523/jneurosci.3935-14.2015.

Goldberg, Y.P., MacFarlane, J., MacDonald, M.L., Thompson, J., Dube, M.P., Mattice, M., et al. (2007). Loss-of-function mutations in the Nav1.7 gene underlie congenital indifference to pain in multiple human populations. *Clin Genet* 71(4)**,** 311-319. doi: 10.1111/j.1399-0004.2007.00790.x.

Klein, C., Wu, Y., Kilfoyle, D., Sandroni, P., Davis, M., Gavrilova, R., et al. (2013). Infrequent SCN9A mutations in congenital insensitivity to pain and erythromelalgia. 84(4)**,** 386-391. doi: 10.1136/jnnp-2012-303719.

Kurban, M., Wajid, M., Shimomura, Y., and Christiano, A.J.D. (2010). A nonsense mutation in the SCN9A gene in congenital insensitivity to pain. 221(2)**,** 179-183. doi: 10.1159/000314692.

Mansouri, M., Elalaoui, S.C., Bencheikh, B.O.A., El Alloussi, M., Dion, P.A., Sefiani, A., et al. (2014). A Novel Nonsense Mutation in SCN9A in a Moroccan Child With Congenital Insensitivity to Pain. *Pediatric Neurology* 51(5)**,** 741-744. doi: 10.1016/j.pediatrneurol.2014.06.009.

Marchi, M., Provitera, V., Nolano, M., Romano, M., Maccora, S., D'Amato, I., et al. (2018). A novel SCN9A splicing mutation in a compound heterozygous girl with congenital insensitivity to pain, hyposmia and hypogeusia. *Journal of the Peripheral Nervous System* 23(3)**,** 202-206. doi: 10.1111/jns.12280.

Nilsen, K., Nicholas, A., Woods, C., Mellgren, S., Nebuchennykh, M., and Aasly, J.J.P. (2009). Two novel SCN9A mutations causing insensitivity to pain. 143**,** 155-158. doi: 10.1016/j.pain.2009.02.016.

Peddareddygari, L., Oberoi, K., and Grewal, R.J.C.r.i.n.m. (2014). Congenital insensitivity to pain: a case report and review of the literature. 2014**,** 141953. doi: 10.1155/2014/141953.

Rajasekharan, S., Martens, L., Domingues, L., and Cauwels, R.J.E.j.o.p.d. (2017). SCN9A channelopathy associated autosomal recessive Congenital Indifference to Pain. A case report. 18(1)**,** 66-68. doi: 10.23804/ejpd.2017.18.01.14.

Ramirez, J.D., Habib, A.M., Cox, J.J., Themistocleous, A.C., McMahon, S.B., Wood, J.N., et al. (2014). NULL MUTATION IN SCN9A IN WHICH NOXIOUS STIMULI CAN BE DETECTED IN THE ABSENCE OF PAIN. *Neurology* 83(17)**,** 1577-1580. doi: 10.1212/wnl.0000000000000913.

Remacle, A., Kumar, S., Motamedchaboki, K., Cieplak, P., Hullugundi, S., Dolkas, J., et al. (2015). Matrix Metalloproteinase (MMP) Proteolysis of the Extracellular Loop of Voltage-gated Sodium Channels and Potential Alterations in Pain Signaling. 290(38)**,** 22939-22944. doi: 10.1074/jbc.C115.671107.

Sawal, H., Harripaul, R., Mikhailov, A., Dad, R., Ayub, M., Jawad Hassan, M., et al. (2016). Biallelic truncating SCN9A mutation identified in four families with congenital insensitivity to pain from Pakistan. 90(6)**,** 563-565. doi: 10.1111/cge.12860.

Shorer, Z., Wajsbrot, E., Liran, T.H., Levy, J., and Parvari, R. (2014). A novel mutation in SCN9A in a child with congenital insensitivity to pain. *Pediatr Neurol* 50(1)**,** 73-76. doi: 10.1016/j.pediatrneurol.2013.09.007.

Staud, R., Price, D., Janicke, D., Andrade, E., Hadjipanayis, A., Eaton, W., et al. (2011). Two novel mutations of SCN9A (Nav1.7) are associated with partial congenital insensitivity to pain. 15(3)**,** 223-230. doi: 10.1016/j.ejpain.2010.07.003.

Sun, J., Li, L., Yang, L., Duan, G., Ma, T., Li, N., et al. (2020). Novel SCN9A missense mutations contribute to congenital insensitivity to pain: Unexpected correlation between electrophysiological characterization and clinical phenotype. *Mol Pain* 16**,** 1744806920923881. doi: 10.1177/1744806920923881.

Yuan, R., Zhang, X., Deng, Q., Si, D., Wu, Y., Gao, F., et al. (2011). Two novel SCN9A gene heterozygous mutations may cause partial deletion of pain perception. 12(10)**,** 1510-1514. doi: 10.1111/j.1526-4637.2011.01237.x.
